# Correlates of Social Exclusion in Social Anxiety Disorder: An fMRI study

**DOI:** 10.1038/s41598-017-00310-9

**Published:** 2017-03-21

**Authors:** Alexandre Heeren, Laurence Dricot, Joël Billieux, Pierre Philippot, Delphine Grynberg, Philippe de Timary, Pierre Maurage

**Affiliations:** 1 Department of Psychology, Harvard University, Cambridge, MA USA; 2 Psychological Science Research Institute, Université Catholique de Louvain, Louvain-la-Neuve, Belgium; 3 Institute of Neuroscience, Université Catholique de Louvain, Brussels, Belgium; 4 Institute for Health and Behavior, Integrative Research Unit on Social and Individual Development (INSIDE), University of Luxembourg, Luxembourg, Luxembourg; 5 Sciences Cognitives et Sciences Affectives (SCALAB), Université de Lille, UMR 9193 Lille, France; 6 Department of Adult Psychiatry, Saint-Luc University Hospital, Brussels, Belgium

## Abstract

Cognitive models posit that social anxiety disorder (SAD) is maintained by biased information-processing vis-à-vis threat of social exclusion. However, uncertainty still abounds regarding the very nature of this sensitivity to social exclusion in SAD. Especially, brain alterations related to social exclusion have not been explored in SAD. Our primary purpose was thus to determine both the self-report and neural correlates of social exclusion in this population. 23 patients with SAD and 23 matched nonanxious controls played a virtual game (“Cyberball”) during fMRI recording. Participants were first included by other players, then excluded, and finally re-included. At the behavioral level, patients with SAD exhibited significantly higher levels of social exclusion feelings than nonanxious controls. At the brain level, patients with SAD exhibited significantly higher activation within the left inferior frontal gyrus relative to nonanxious controls during the re-inclusion phase. Moreover, self-report of social exclusion correlates with the activity of this cluster among individuals qualifying for SAD diagnosis. Our pattern of findings lends strong support to the notion that SAD may be better portrayed by a poor ability to recover following social exclusion than during social exclusion *per se*. These findings value social neuroscience as an innovative procedure to gain new insight into the underlying mechanisms of SAD.

## Introduction

With a lifetime prevalence of more than 12%, social anxiety disorder (SAD) is among the most common anxiety disorders^1^. Current diagnostic manuals defined SAD as intense fear and avoidance of evaluative scrutiny in a wide range of interpersonal situations that may lead to negative evaluation and social exclusion^2, 3^. SAD is associated with considerable distress and impaired daily functioning^4, 5^. Moreover, SAD follows a chronic debilitating course if untreated^6^.

A curious feature of SAD is that it persists even when sufferers perform naturalistic exposure to at least some feared social situations on a regular basis in their daily life^7^. As argued by prominent cognitive theorists of SAD^8–10^, one possibility is that people with chronic SAD process cues that signal potential social exclusion in ways that maintain their anxiety^3^. Accordingly, laboratory studies involving probe detection and probe discrimination task indicate that people with SAD respond faster to probes replacing cues denoting potential social exclusion, such as faces expressing social disapprobation (e.g., anger or contemptuous disgust), than probes replacing neutral cues, thereby exhibiting a selective bias vis-à-vis threat of social exclusion that is absent in nonanxious individuals (for a meta-analysis, see ref. 11). Moreover, these biases may play an important role in the maintenance of the SAD^12–14^. Yet, uncertainty still abounds regarding the impact of social exclusion in SAD.

For identifying the correlates of social exclusion, several experimental paradigms were designed; however, the Cyberball task is by far the most efficient and widely used^15–19^. During this task, participants are told that they are playing an online ball-tossing game with two other partners^17–19^. Actually, there are no real other players, participants play with computer-guided players. The Cyberball task includes “inclusion” phases during which the two other partners play with the participants, and “exclusion” phases during which they throw the ball only to each other, thus excluding the participant^16–19^. The comparison of exclusion versus inclusion phases is assumed to capture the *sensitivity to social exclusion*. Accordingly, this paradigm has been able to robustly induce genuine self-report feelings of social exclusion among healthy volunteers^16, 20–25^. Likewise, neuroimaging studies have reported that several regions are engaged during social exclusion, including the dorsal anterior cingulate cortex (dACC), insula, middle frontal gyrus (MFG), inferior temporal gyrus (IFG), parahippocampal gyrus (PHG), thalamus, and other portions of the ACC (e.g., posterior cingulate cortex; PCC)^16, 20–25^.

Although the aforementioned research efforts to advance the understanding of social exclusion, only a couple of studies have investigated how sensitivity to social exclusion relates to SAD. At the behavioral level, individuals with SAD exhibit higher self-report distress and more prolonged recovery following the exclusion phase of the Cyberball^26, 27^. Likewise, individuals with elevated social anxiety differ from nonanxious control participants in their ability to self-regulate following exclusion^27, 28^. For instance, social anxiety has been associated with a pattern of vocal insecurity in the production of command utterances following the exclusion phase^28^.

Apart from behavioral studies, brain correlates of social exclusion have been studied to an extremely limited extend in SAD^29^. Actually, to the best of our knowledge, only one study investigated the impact of social anxiety on brain responses to social exclusion^30^. In their study, Nishiyama *et al.* ^30^ explored how social support can mitigate social distress associated with social exclusion. To explore this issue, nonanxious undergraduates were initially included and later excluded from a modified version of the Cyberball. In the latter half of the exclusion block, participants were provided with supportive messages. The authors reported a significant positive correlation between self-reported fear of negative evaluation (as a proxy of social anxiety) and activations in the right MFG elicited by the exclusion Cyberball’s phase. Yet, as Nishiyama *et al.* ^30^ included supportive messages during the exclusion phase, it remains particularly difficult to interpret their findings in light of the previous behavioral studies reporting that social anxiety is better portrayed by a poor ability to recover and self-regulate following social exclusion than during the social exclusion *per se* ^26–28^.

Moreover, all the aforementioned studies were conducted among healthy undergraduates with elevated levels of social anxiety. Although many people experience symptoms of social anxiety (e.g., fear of public speaking) without qualifying for SAD^31^, people qualifying for the diagnosis of SAD exhibit selective cognitive bias vis-à-vis cues that signal potential social exclusion that are absent in people free of the diagnosis^11^. Moreover, several studies involving other experimental paradigms have reported different neural between individuals qualifying for SAD diagnosis and healthy participants^29, 32^. As concerns vis-à-vis social exclusion is a hallmark feature of SAD diagnosis in the current diagnostic manuals^2, 3^, it remains decisive to explore the impact of social exclusion among individuals qualifying for SAD diagnosis.

**Figure 1:**
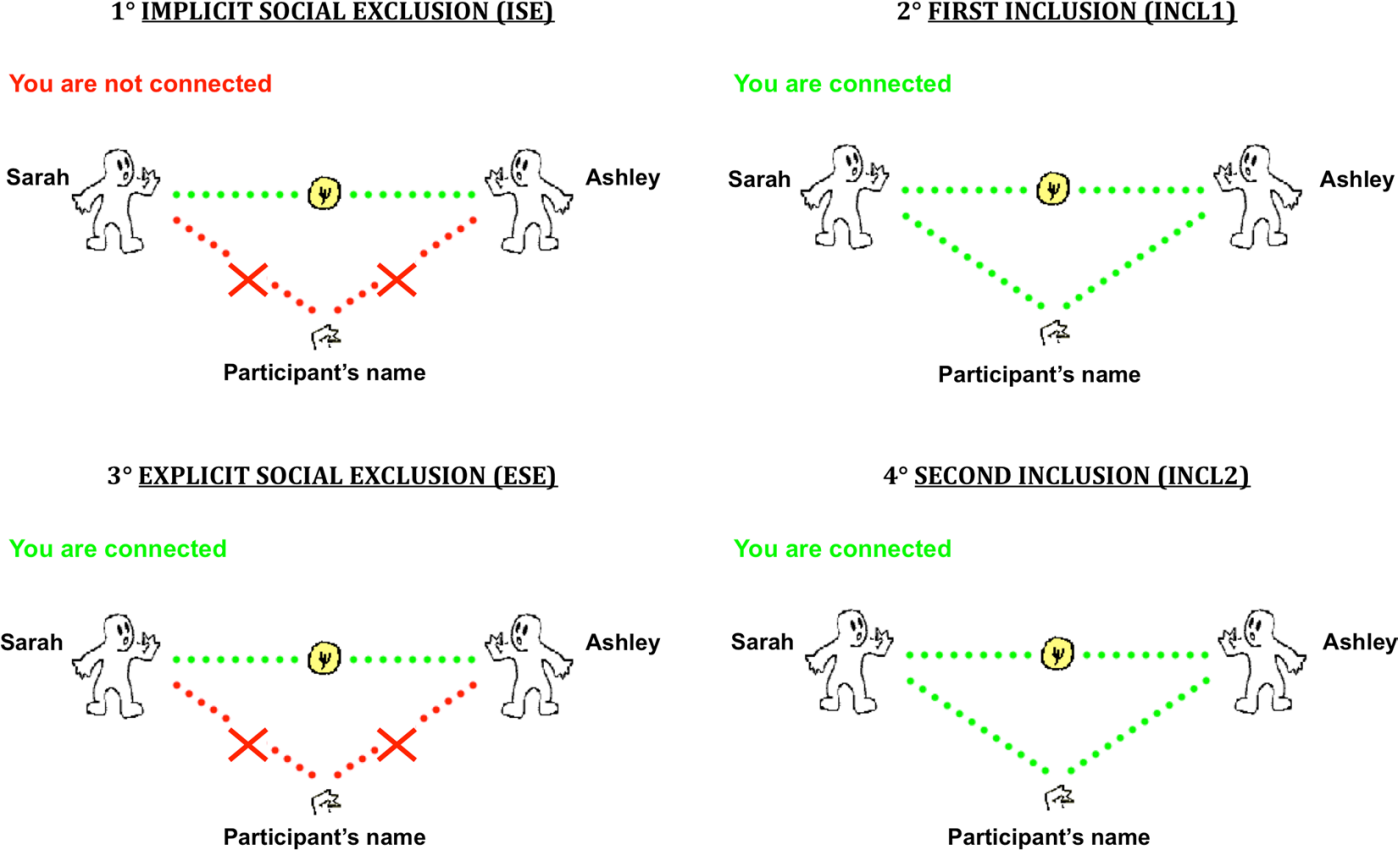
Illustration of the four successive experimental conditions: (1) Implicit social exclusion (*ISE*); (2) First inclusion (*INCL1*); (3) Explicit social exclusion (*ESE*); (4) Second inclusion i.e., re-inclusion after social exclusion (*INCL2*). Note. The illustration (including the little man) has been drawn, following initial Cyberball’s studies [see refs 16–19], by Pierre Maurage^33^ who co-authored this paper.

In the current study, for the first time, we thus examined the self-report and neural correlates of social exclusion among female participants with a DSM-5 SAD diagnosis^2^ as compared to matched nonanxious control participants (NACPs). Participants were presented with a modified version of the Cyberball paradigm including four successive conditions (Fig. 1): (a) implicit social exclusion (*ISE*), where participants were told that the intranet connection was not effective yet because of technical problems, but that they could watch the two other participants already playing; (b) first inclusion (*INCL1*), where participants were told they were connected and played with other players; (c) explicit social exclusion (*ESE*), where participants received five throws and were then excluded from the game (i.e., other players started playing exclusively together, participant never receiving the ball anymore); and (d) second inclusion (*INCL2*), where participants were connected and re-included in the game. This fourth condition was added here, following earlier studies^33^ to explore the brain correlates of re-inclusion after exclusion. Finally, following previous fMRI studies using the Cyberball^16, 33^, we included a post-manipulation check assessment of exclusion feelings.

Of primary interest were the differences between individuals with SAD and NACPs in behavioral and brain responses to the exclusion phase. As earlier behavioral studies indicated that individuals with SAD exhibited longer recovery following the exclusion phase^26–28^, we hypothesized that those individuals should show larger behavioral and brain differences than NACPs during social re-inclusion after exclusion ends. Yet, given that this study is the first of its kind, it remained particularly difficult to formulate precise hypothesis including specific brain areas, so that we conducted whole-brain analyses. At the behavioral level, we expected higher self-report feelings of social exclusion among individuals with SAD relative to NACPs.

## Results

### Participants

We recruited 23 right-handed female participants with a primary DSM-5 diagnosis of SAD^2^. SAD participants were matched for age (+/−2 years) and education level with 23 matched NACPs who were free of SAD symptoms and of any history of psychiatric disorders. As shown in Table 1, individuals with SAD and NACPs were indistinguishable in terms of age and years of education, thus confirming the correct matching between groups. Nevertheless, although the two groups did not significantly differ on state-anxiety, individuals with SAD showed higher scores than NACPs for depressive symptomatology and trait-anxiety, thereby supporting the clinical status of our sample.

**Table 1:** Demographic and clinical measures for patients qualifying for the DSM-5 diagnosis of social anxiety disorder (SAD) and matched nonanxious control participants (NACP): mean (*SD*).

	SAD (N = 23)	NACP (N = 23)	*t* (44) or *χ*²	*p*
Demographic measures				
Age	24.96 (6.46)	25.30 (5.62)	.20^a^	.35
Educational level	13.30 (1.92)	13.39 (2.11)	.15^a^	.88
Clinical measures				
Beck Depression Inventory (BDI-II)	15.70 (8.43)	7.70 (5.48)	3.81^a^	<0.01
State and Trait Anxiety Inventory - Trait (STAI-T)	51.04 (9.26)	39.65 (8.26)	4.39^a^	<0.01
State and Trait Anxiety Inventory - State (STAI-S)	34.74 (8.21)	31.65 (7.65)	1.32^a^	.19
Liebowitz Social Anxiety Scale (LSAS)	74.22 (11.11)	28.04 (13.73)	12.54^a^	<0.01

Note. Education level was assessed according to the number of years of education completed after starting primary school.

### Behavioral results

**Figure 2:**
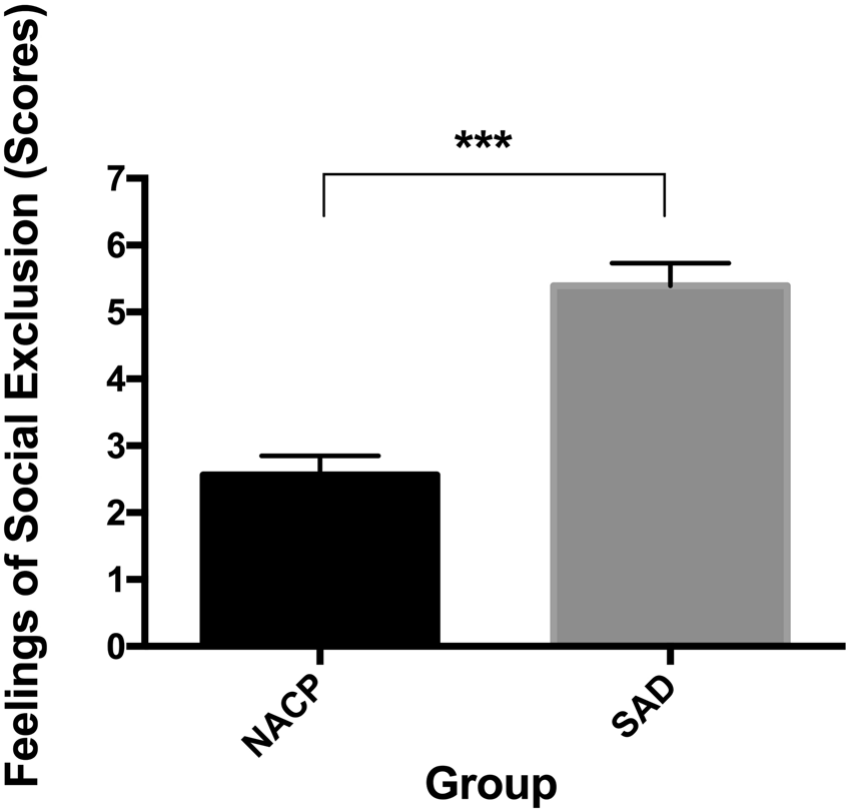
Self-report post-manipulation feelings of social exclusion scores for individuals qualifying for the diagnosis of social anxiety disorder (SAD) and matched nonanxious control participants (NACP). Error bars represent standard errors of the mean; ***p < 0.001.

Both SAD and NACPs reported feelings of social exclusion significantly higher than the minimal score indexing no feelings of exclusion, *t*(22) = 15.73, *p* < 0.001 and *t*(22) = 8.94, *p* < 0.001, respectively. These finding indicated that the task worked as intended to induce genuine self-report feelings of social exclusion. Moreover, SAD exhibited significantly higher scores than NACPs, *t*(44) = 6.32, *p* < 0.001 *(*Fig. 2).

Likewise, Liebowitz Social Anxiety Scale^34^ (LSAS) scores were positively correlated with the intensity of the self-report feelings of social exclusion in SAD (*r* = 0.72, *p* < 0.01), but not in NACPs (*r* = 0.18, *p* = 0.40). This observation remained significant when controlling for Beck Depression Inventory^35^ (BDI-II) and trait-anxiety^36^ (STAI-Trait) scores (*r* = 0.76, *p* < 0.01 for SAD; *r* = 0.12, *p* = 0.61 for NACPs).

### Functional Imaging Results

#### Social exclusion activation patterns

For NACPs, one-sample *t*-tests vis-à-vis the *ESE-ISE* contrast showed significant clusters (after correction for false discovery rate) of activated voxels within the left dACC, left insula, thalamus, and right PCC and supramarginal gyrus (Table 2a). Similarly, one sample *t*-tests indicated that SAD showed a very similar pattern of significant clusters (after correction for false discovery rate), including the dACC, the bilateral insula, the bilateral thalamus, and the PCC (Table 2a). As shown in Table 2c, corrected between-group *t*-values indicated that these brain activations did not differ between SAD and NACPs.

**Table 2:** Significant brain activations for (a) social exclusion as compared to implicit exclusion (*ESE – ISE*) among matched nonanxious control participants (NACP) and individuals with qualifying for the diagnosis of Social Anxiety Disorder (SAD); (b) re-inclusion after social exclusion as compared to first inclusion (*INCL2 – INCL1*) in each group; (c) group comparisons during social exclusion and re-inclusion.

(a) ESE-ISE									
Group	Brain area	Talairach coordinates (mm)			BA	Side	k	*t* ^a^	
		*x*	*y*	*z*					
NACP	dACC	−1	−20	36	24	L	485	8.76	
	Thalamus	−7	−15	10		L	58	6.13	
	Insula	−43	15	−2	13	L	41	7.05	
	SMG	46	−47	27	40	R	30	7.35	
	PCC	10	−55	4	30	R	26	5.46	
SAD	dACC	−23	−17	36	24	L	511	7.85	
	Insula	−37	−8	3	13	L	48	6.40	
	Thalamus	−13	−17	6		L	47	5.50	
	PCC	−10	−44	27	23	L	27	4.42	
	SMG	55	−41	25	40	R	21	5.41	
	Insula	41	12	0	13	R	24	6.51	
**(b) INCL2-INCL1**									
**Group**	**Brain area**	**Talairach coordinates (mm)**			**BA**	**Side**	***k***	***t*** ^a^	
		***x***	***y***	***z***					
**NACP**	dACC	−1	4	42	24	L	129	9.02	
	MFG	−19	−8	56	6	L	64	8.95	
	SMG	50	−48	19	40	R	32	8.24	
	Thalamus	−13	−17	12		L	26	8.40	
	Insula	−40	−8	3	13	L	25	7.87	
	ITG	−50	−66	0	37	L	23	8.43	
**SAD**	dACC	−9	−10	35	24	L	112	7.93	
	MFG/IFG	−32	7	33	6/9	L	125	8.85	
	Thalamus	−14	−15	0		L	21	8.73	
	Insula	−43	3	3	13	L	22	8.09	
	Anterior Lobe (Cerebellum)	−34	−53	−24		L	24	8.75	
	ITG	−50	−62	0	37	L	22	7.54	
**(c) Group comparison**									
**Contrast**	**Comparison**	**Brain area**	**Talairach coordinates (mm)**			**BA**	**Side**	***k***	***t*** ^**b**^
			**x**	**y**	**z**				
**ESE - ISE**	**SAD > NACP**			No significant activation					
	**SAD < NACP**			No significant activation					
**INCL2 - INCL1**	**SAD > NACP**	IFG (pars opercularis)	−40	1	30	9	L	24	16.18
	**SAD < NACP**			No significant activation					

Notes. x, y, and z are Talaraich stereotaxic coordinates of peak-height voxel-cluster activations.

BA = Brodmann’s area; dACC = dorsal anterior cingulate cortex; IFG = inferior frontal gyrus; ITG = inferior temporal gyrus; k = cluster size in units of contiguous voxels; L = left hemisphere; MFG = middle frontal gyrus; PCC = posterior cingulate cortex; R = right hemisphere; SMG = supramarginal gyrus. Threshold set at *p < *0.001, p-values are corrected for multiple comparisons using a Monte-Carlo simulation (iterations = 10,000) cluster size threshold adjustment, with a minimum cluster size of 20 contiguous voxels for the one-sample *t*-tests and 16 contiguous voxels for the two-sample *t*-tests. ^a^Value for *t*(22); ^b^Value for *t*(44).

#### Persistence of social exclusion activation patterns

**Figure 3:**
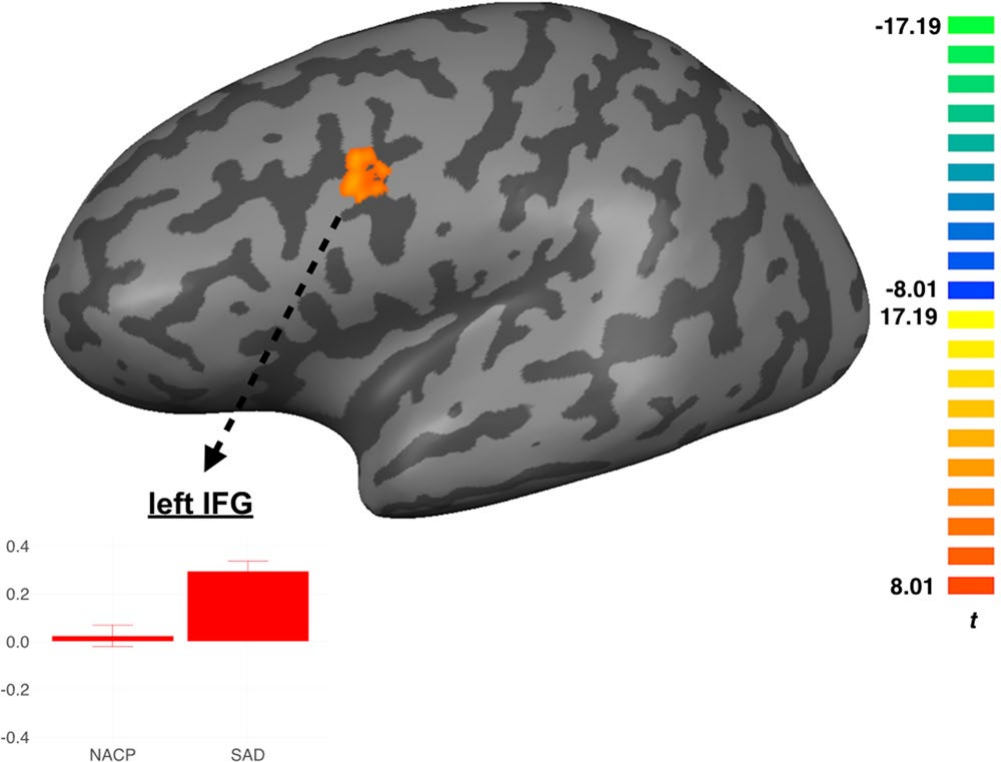
Group comparison for the re-inclusion contrast (*INCL2 – INCL1*) between individuals qualifying for the diagnosis of social anxiety disorder (SAD) and matched nonanxious control participants (NACP). Threshold set at *p* < 0.001, *p*-values are corrected for multiple comparisons using a Monte-Carlo simulation (iterations = 10,000) cluster size threshold adjustment, with a minimum cluster size of 16 voxels for the two-sample *t*-tests. Beta-values for each group are presented in the bar plot. Error bars represent standard errors of the mean.

For NACPs, one-sample *t*-tests vis-à-vis the *INCL2-INCL1* contrast revealed significant clusters of activation within the left dACC, MFG, thalamus, insula, inferior temporal gyrus, as well as within the right supramarginal gyrus (Table 2b). Likewise, individuals with SAD exhibited a very similar pattern of clusters, including MFG, dACC, thalamus, insula, and IFT (Table 2b). As shown in Fig. 3, corrected between-group *t*-values revealed that SAD, as compared to NACPs, culminated in higher activations within the left IFG, and especially the pars opercularis of the IFG, for the *INCL2-INCL1* contrast.

Complementary region-of-interest (ROI) analyses^37^ indicated that the significant cluster of contiguous voxels extracted from the *INCL2-INCL1* contrast was significantly activated in each group (*t* = 31.68, *p* < 0.00001, for SAD, *t* = 4.181, p < 0.0001 for NACPs), ensuring that the significant corrected between-group effect vis-à-vis the *INCL2-INCL1* contrast did not merely mirror a decreased activation among the NACPs following exclusion. Moreover, the significant *INCL2-INCL1* cluster was also significantly activated among each group vis-à-vis the *ESE-ISE* contrast (*t* = 22.38, *p* < 0.0001, for SAD; *t* = 21.73, *p* < 0.0001, for NACPs). Although the ROI was equally activated in both groups for the *ESE–ISE* contrast, this latter analysis suggests that individuals with SAD exhibit a genuine persistence of activations within this cluster during the re-inclusion. Finally, neither the contrasts comparing inclusion conditions with the non-inclusion baseline (i.e., *INCL1-ISE*, *INCL2-ISE*) nor the *INCL2-ESE* and *INCL1-ESE* contrasts yielded significant patterns of activations.

#### Brain-behaviors correlations vis-à-vis the persistence of social exclusion

**Figure 4:**
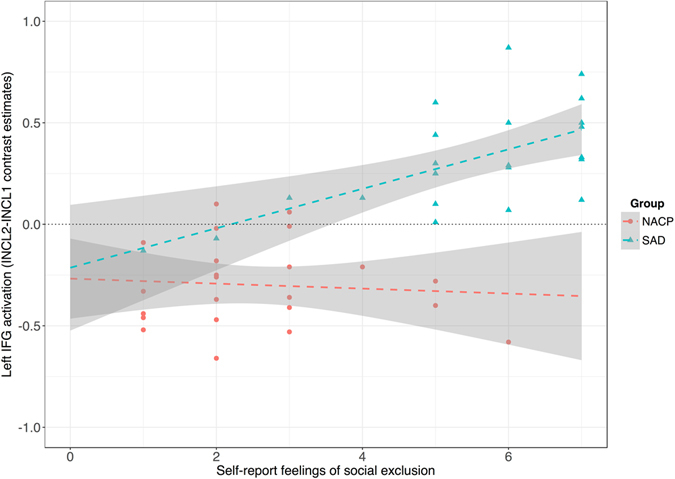
Pearson correlations between the self-report post-manipulation feelings of social exclusion scores and activations within the left inferior frontal gyrus (IFG) for the *INCL2-INCL1* contrast among individuals qualifying for the diagnosis of social anxiety disorder (SAD) and matched nonanxious control participants (NACPs). The grey zones represent the 95% confidence interval of the linear model.

The intensity of the self-report feelings of social exclusion was positively correlated with the left IFG activation (*INCL2-INCL1* contrast estimates) in SAD (*r* = 0.47, *p* < 0.01) but not in NACPs (*r* = −0.09, *p* = 0.71) (Fig. 4).

#### Correlations between imaging and clinical data

The intensity of the LSAS was positively correlated with left IFG activation (*INCL2-INCL1* contrast estimates) in SAD (*r* = 0.46, *p* < 0.02) but not in NACPs (*r* = 0.20, *p* = 0.31). Although neither BDI-II (*r* = −.08, *p* = 0.73) nor STAI-Trait scores correlated (*r* = 0.07, *p* = 0.76) with the *INCL2-INCL1* contrast estimates in SAD, the correlation became even larger when controlling for BDI-II and STAI-Trait scores (*r* = 0.54, *p* < 0.01) among those participants. None of these correlations reached significance level among NACPs (*r* = 0.30, *p* = 0.17 for the BDI-II; *r* = 0.25, *p* = 0.24 for the STAI-Trait; *r* = 0.11, *p* = 0.65 when controlling for BDI-II and STAI-Trait scores).

## Discussion

The primary purpose of this study was to answer two major questions. First, do individuals with SAD exhibit different self-report and neural responses to social exclusion as compared to NCAPs? Indeed, although researchers have seldom investigated the association of social anxiety with behavioral and brain correlates of social exclusion among healthy undergraduate students, no previous study had included a sample of individuals with SAD. Second, as outlined in earlier behavioral studies^26–28^, we sought to further examine whether SAD is best characterized by impoverished ability to recover following the exclusion phase than disruption in the ability to self-regulate during the exclusion *per se*.

Consistent with our hypothesis, individuals with SAD demonstrated significant differences in both self-report and neural responses to social exclusion relative to NACPs. At the behavioral level, although we replicated previous observation indicating that the Cyberball paradigm is indeed able to induce genuine self-report feelings of social exclusion^16–19^, we demonstrated, for the first time, that this effect is stronger among individuals qualifying for DSM-5 diagnosis of SAD relative to those free of the diagnosis. At the brain level, although we substantiated the typical patterns of activations (i.e., dACC, insula, MFG, PCC)^16, 20–25^ coupled with the different Cyberball’s phases in each group, individuals qualifying for SAD diagnosis exhibited significantly higher activation within the left IFG, and especially within the pars opercularis of the IFG, than those free of the diagnosis during the re-inclusion phase. Taking us one step further, self-report feelings of social exclusion also positively correlated with the left IFG activation in SAD. Notably, no group-differences were found when isolating the cerebral correlates of social exclusion *per se*, that is *ESE-ISE*. Altogether, these findings are in keeping with earlier studies conducted among undergraduate volunteers that indicate that social anxiety is associated with difficulty to self-regulate following exclusion^26–28^; extending this observation, for the first time, to individuals qualifying for the diagnosis of SAD.

From a theoretical point of view, our findings dovetail with Clark and Wells^38^ prominent model of SAD, in which they posit that individuals with SAD exhibit delayed recovery following socially threatening event, such as exclusion. Interestingly, according to this model, it is assumed that this delayed recovery results from postmortem ruminative processing of the event that, in turn, impairs emotion regulation. Although we did not assess postmortem rumination, this shift away from the Cyberball’s literature to rumination is especially notable, given previous observations that rumination has been associated with activation within the left IFG, and particularly the pars opercularis (Broca’s area)^39, 40^. Likewise, in line with earlier works indicating that self-referential thought processing involves language production^41, 42^, IFG activation has previously been reported in neuroimaging studies investigating inner speech^43, 44^, and especially inner speech during self-related processing^44^. Although this possibility remains speculative, our findings suggest that SAD researchers should draw on these conceptual bridges and more closely examine the role of IFG in SAD.

Interestingly, our findings are at odds with Nishiyama *et al*.^30^ who reported a significant positive correlation between self-report social anxiety symptoms and activations in the right MFG following exclusion. However, as mentioned before, these authors relied on a somewhat different inclusion phase in which participants received emotionally supportive notifications during the re-inclusion phase. It thus remains particularly difficult to properly equate their findings to ours. Moreover, their participants were healthy undergraduates. On the other hand, Nishiyama *et al*.’s findings^30^ indicated that social anxiety did not correlate with brain activations elicited by the differences between inclusion and exclusion phases, substantially endorsing our absence of group-difference vis-à-vis the exclusion phase. Nonetheless, the findings reported here should be regarded only as the first study examining the self-report and neural correlates of social exclusion among individuals qualifying for the DSM-5 diagnosis of SAD. Our analyses were thus primarily exploratory. ROI-based replications in new clinical samples will ultimately be required to effectively unravel the actual role of IFG and MFG during social exclusion and re-inclusion in SAD.

Our findings may also yield therapeutic implications. By improving self-regulation during and following social exclusion, clinicians may foster beneficial cascade of downstream benefits as it effects may propagate throughout IFG activation during the re-inclusion phases and the post-event feelings of social exclusion. Likewise, the present findings reveal postmortem of the exclusion as a promising target whose therapeutic intervention may produce beneficial downstream impacts. Programs have already been developed to improve postmortem of the event in SAD^45^. Likewise, mindfulness training improves one’s ability to regulate postmortem ruminative processes in SAD^46^. In addition, as concerns vis-à-vis threat of social exclusion appear to be among the hallmark features of SAD^2, 3^, future studies should also explore how intervention affects the present pattern of findings.

The present study has some limitations. First, the cross-sectional nature of this study prevents us from characterizing observed neural patterns as causally related to development and maintenance of the disorder over time. Longitudinal work would thus be useful in delineating the precise relationship between persistence of exclusion feelings and brain function over the lifespan. Second, we only included female participants to control for potential gender effect. On the other hand, SAD yields both greater illness burden and higher prevalence rates in women than in men^47, 48^. Third, our manipulation check scale was less than ideal as it targets the presence of exclusion feelings after the re-inclusion. Although this decision was based upon previous studies^33^, future studies might assess the presence of exclusion feeling directly after the completion of each Cyberball’s phase. On the other hand, such a modification may alter the credibility of the cover story. Fourth, participants were administrated the Cyberball’s phases in a fixed sequential order. Yet, Cyberball’s potency constraints the administration of the inclusion phase prior to the exclusion phase^17–19^. Next steps would thus be to replicate the present study using different research paradigms allowing randomized block presentation. Fifth, we did not assess the participants’ credibility of the cover story. However, it is worth noting that feelings of social exclusion following Cyberball can even be elicited when the participants knew that they were playing with computer-generated co-players^49^. Finally, although cluster-extend based thresholding has a relatively high sensitivity in small sample sizes^50^, it has low spatial specificity when clusters are large^51, 52^. On the other hand, our clusters were small-to-medium sized as compared to previous studies using a similar approach^53, 54^.

## Methods

### Participants’ selection

SAD patients were recruited via private practitioners and through local community listservs in the French-speaking part of Belgium. Exclusion criteria were: (a) the absence of DSM-5 criteria^2^ for SAD, (b) the presence of additional psychiatric disorders, (c) current or past heart, respiratory, or neurological problems, (d) current pharmacological treatments, (e) presence of metallic foreign particles or a cardiac pacemaker, (f) pregnancy at the time of the testing, and (g) insufficient knowledge of French language. These criteria were checked through a medical interview and by using the Mini International Neuropsychiatric Interview^55^ (MINI). A PhD level clinical psychologist completed all the interviews.

Participants were also administered the LSAS^34^. Each participant met the SAD criteria at the MINI and scored above 56 on the LSAS (i.e., the cut-off score for probable diagnosis of SAD in the French version of the scale^56^). NACPs were recruited through the volunteer pool of the Université catholique de Louvain (Belgium). Their absence of current and past psychiatric symptomatology was checked using the MINI. All participants also completed the Spielberger State and Trait Anxiety Inventory^36^ (STAI-State and –Trait) as well as the BDI-II^35^. We used the validated French versions of these scales^35, 57, 58^.

### Task and Procedure

Blood oxygenation level-dependent (BOLD) signal was recorded during the Cyberball task^17–19^. Participants were told that this experiment was about mental imagery and that the task was used to help them visualizing other players. This cover story ensured that participants believed the other players were real^16–19^. In the scanner, participants saw an animated ball-tossing game, with an icon representing their own hand at the bottom and the two other players depicted as animated icons in the upper corners. Players’ names were written besides each player’s icon^33^. The Intranet connection status (“you are not connected” for the first condition; “you are connected” for the three others) was written in the top left part of the screen. When receiving the ball, participants had 2500 ms to choose (using a two-button response pad) which player they wanted to give the ball to. As mentioned above, each participant took part in four successive conditions (see Fig. 1): *ISE*, *INCL1*, *ESE*, and *INCL2* (see the closing section of the introduction). Except for the *ISE–INCL1* switch (i.e., from “not connected” to “connected”), participants were not informed of the transition between the successive conditions. Each condition lasted for 125 s (50 brain volumes). Computer players’ speed varied randomly between 500 and 2000 ms, and was adapted to obtain 100 throws per condition (e.g., *ISE*). If the participant did not throw the ball within 2500 ms, it was automatically thrown to a random player. The first 10 volumes of each condition were excluded from analyses (which relied on 40 volumes per condition) to avoid overlap between the activations associated with each condition.

The two main experimental contrasts were: (a) *ESE–ISE*, isolating the cerebral correlates of social exclusion feelings, as *ESE* and *ISE* are perceptually identical (i.e., other players excluding the participant, who never receives the ball), but differ for social exclusion: in *ISE*, participant knows she is not participating because of technical reasons, whereas in ESE, she is explicitly excluded by other players, eliciting exclusion feelings; (b) *INCL2–INCL1*, exploring the persistence of exclusion feelings after exclusion ends. *INCL1* and *INCL2* are perceptually identical (i.e., the participant being included, with a 50% probability of getting the ball from another player), but in *INCL1* the participant has not yet been explicitly excluded by others, whereas *INCL2* is just following the *ESE* condition. Finally, likewise previous fMRI studies using the Cyberball^16, 33^, we included a post-experimental manipulation check to ensure the presence of exclusion feelings, as participants answered (after the last condition) a post-manipulation check scale assessing social exclusion (i.e. “*I felt excluded by other participants*”) and anchoring from 1 (*Absolutely not*) to 7 (*Extremely*). The whole testing session lasted about 45 minutes per participant.

### Ethical Consideration

The study was approved by the ethical committee of the Université catholique de Louvain (Belgium) and carried out according to the Declaration of Helsinki. Participants provided their written informed consent prior to the study. After the experiment, they were fully debriefed and received compensation (25 euros). Debriefing consisted of information about the exact nature of the study. This way, participants were also told that they had played the game with two computer-generated confederates, not real players.

### Imaging Acquisition Parameters

Imaging was performed using a 3 Tesla MR scanner (Achieva, Philips Healthcare®, Best, The Netherlands) and a 32 channels phased array head coil. Anatomical scan of the whole brain was provided by a 3D fast T1-weighted gradient echo sequence with an inversion prepulse [Turbo field echo, time of repetition (TR) = 9 ms, time of echo (TE) = 4.6, flip angle (FA) = 8°, 150 slices—thickness = 1 mm, field of view (FOV) = 220 × 197 mm^2^ giving an in-plane resolution = 0.81 × 0.95 mm^2^. The SENSE factor (parallel imaging) was set to 1.5]. Blood oxygen level dependent (BOLD) fMRI data were acquired using a 2D single shot T2*-weighted gradient echo-planar imaging sequence (TR = 2250 ms, TE = 27 ms, FA = 85°, FOV = 220 × 220 mm^2^, scan resolution = 80 × 80, slice thickness = 3 mm with no interslice gap, and SENSE factor = 2.5). Each volume comprised 41 axial slices acquired in ascending interleaved sequence. Recording comprised one 208-volume run (50 volumes per condition, interleaved by 2 volumes transition periods). Foam pads restrained the head during both the anatomical and functional MRIs.

### Data Analytic Approach

#### Preprocessing

Data analysis was performed using the BrainVoyager QX® Version 2.3.1. (Brain Innovation, Maastricht, The Netherlands). The volumes were corrected to minimize effects of slice scan time correction (cubic spline interpolation) and head movements (3D motion correction with trilinear sinc interpolation) was applied. After correction, none of the data included in the study exceeded motion of 2 mm in any given axis or had spike-like motion of more than 1 mm in any direction during a given fMRI session. Further preprocessing comprised spatial smoothing through Gaussian filter of 5 mm full-width half-maximum (FWHM) isotropic Gaussian kernel, temporal filtering (linear trend removal), and correction for serial correlations. The anatomical and functional data sets of each subject were co-registered and the resulting matching brain images were fit to standardized Talairach space, with a resulting voxel size of 3 × 3 × 3 mm.

#### fMRI Statistical analysis

At the first-level, fixed-effect analysis was conducted for each participant with four predictors created for the Cyberball conditions. The expected BOLD signal change for each predictor was modeled by a hemodynamic response function (two-gamma HRF). A multiple linear regression of the signal time course at each voxel was calculated. Voxel-wised statistical maps were generated and predictor estimates (beta weights) were computed for each individual at a whole-brain level. Then, predictor estimates of the two contrasts of interest (*ESE–ISE* and *INCL2–INCL1*) were analyzed into a second-level whole-brain general linear model with subjects treated as a random effect (i.e., random effect analysis) and group as between-subject factor. Within-group contrast-based comparisons were performed using whole-brain one-sample *t*-tests and between-group contrast-based comparisons were performed using two-sample *t*-tests.

To correct for multiple comparisons, we used a cluster-extent based thresholding approach^50, 51, 59, 60^. This approach consists of two stages. First, a pre-determined voxel-level *primary threshold* defines clusters by retaining groups of suprathreshold voxels. Second, a cluster-level *extent threshold*, measured in units of contiguous voxels (*k*), is determined based on the estimated distribution of cluster sizes under the null hypothesis of no activation in any voxel in that cluster. The sampling distribution of the largest null cluster size under the global null hypotheses of no signal is typically estimated using theoretical methods (e.g., random field theory, Monte Carlo simulation). In contrast to voxel-level correction, this approach has relatively high sensitivity in small sample sizes (N < 50)^50^. Moreover, it accounts for the fact that individual voxel activations are not independent of the activations of their neighboring voxels^50, 61, 62^. Following Woo *et al*.^50^, we set the primary voxel-wise threshold at *p* < 0.001 (for both one- and two-sample *t*-tests). Thresholded maps were then submitted to a cluster size threshold adjustment based on the estimates of the map’s spatial smoothness and on a Monte Carlo simulation procedure (10,000 iterations) to estimate cluster level false-positives rates. It resulted in a minimum cluster size of 20 contiguous voxels (540 mm^3^) for the one-sample *t*-tests and 16 contiguous activated voxels (432 mm^3^) for the two-sample *t*-tests.

To further depict the signal behind between-group effects across conditions, we performed ROI-based analyses on the significant clusters activations for each group separately^37^. Moreover, for each corrected brain activation map, correlational analyses were performed among contrast-based beta weights of the activated voxel-clusters and the severity of SAD as well as the intensity of the self-report post-manipulation feelings of social exclusion. Likewise, we also explored potential influence of depressive symptoms and trait-anxiety using a similar procedure^62^.

## References

[1] Kessler RC (2005). Lifetime prevalence and age-of-onset distributions of DSM-IV disorders in the national comorbidity survey replication. Arch Gen Psychiat.

[2] American Psychiatric Association. *Diagnostic and statistical manual of mental disorders* (5^th^ ed.) (American Psychiatric Publishing, 2013).

[3] World Health Organization. ICD-10 Classifications of Mental and Behavioural Disorder: Clinical Descriptions and Diagnostic Guidelines (1992).

[4] Kessler RC (2003). The impairments caused by social phobia in the general population: Implications for intervention. Acta Psychiat. Scand..

[5] Schneier FR (1992). Social Phobia: Comorbidity and Morbidity in an Epidemiologic Sample. Arch Gen Psychiat.

[6] Steinert C, Hofmann M, Leichsenring F, Kruse J (2013). What do we know today about the prospective long-term course of social anxiety disorder?A systematic literature review. J Anxiety Disord.

[7] Hirsch CR, Clark DM (2004). Information-processing bias in social phobia. Clin Psychol Rev.

[8] Rapee RM, Heimberg RG (1997). A cognitive-behavioral model of anxiety in social phobia. Behav Res Ther.

[9] Heimberg, R. G., Brozovich, F. A., & Rapee, R. M. A cognitive model of social anxiety disorder: Update and extension. In *Social anxiety: Clinical, developmental, and social perspectives — Second edition* (ed. Hofmann, S. G. & DiBartolo, P. M.) 395–422 (Academic Press, 2010).

[10] Wong QJJ, Rapee RM (2016). The aetiology and maintenance of social anxiety disorder: A synthesis of complimentary theoretical models and formulation of a new integrated model. J Affect. Disord..

[11] Bantin T, Stevens S, Gerlash AL, Hermann C (2016). What does the facial dot-probe task tell us about attentional processes in social anxiety?. J Behav Ther Exp Psychiatry.

[12] Hallion LS, Ruscio AM (2011). A meta-analysis of the effect of cognitive bias modification on anxiety and depression. Psychol Bull..

[13] Heeren A, Mogoaşe C, Philippot P, McNally RJ (2015). Attention bias modification for social anxiety: A systematic review and meta-analysis. Clin Psychol Rev.

[14] Heeren A, Peschard V, Philippot P (2012). The causal role of attentional bias to threat cues in social anxiety: A test on a cyber-ostracism task. Cognit Ther Res.

[15] Bolling DZ (2011). Development of neural systems for processing social exclusion from childhood to adolescence. Dev Sci.

[16] Eisenberger NI, Lieberman MD, Williams KD (2003). Does rejection hurt?An fMRI study of social exclusion. Science.

[17] Williams KD, Cheung CKT, Choi W (2000). CyberOstracism: Effects of being ignored over the Internet. J Pers Soc Psychol.

[18] Williams KD, Jarvis B (2006). Cyberball: A program for use in research on ostracism and interpersonal acceptance. Behav Res Methods Instrum Comput.

[19] Williams KD (2007). Ostracism. Annu Rev Psychol.

[20] Dewall CN (2010). Acetaminophen reduces social pain: behavioral and neural evidence. Psychol Sci..

[21] Gunther-Moor B (2012). Social exclusion and punishment of excluders: neural correlates and developmental trajectories. Neuroimage.

[22] Kross E, Egner T, Ochsner K, Hirsch J, Downey G (2007). Neural dynamics of rejection sensitivity. J Cognitive Neurosci.

[23] Lieberman MD, Eisenberger NI (2009). Pains and pleasures of social life. Science.

[24] Onoda K (2010). Does low self-esteem enhance social pain?The relationship between trait self-esteem and anterior cingulate cortex activation induced by ostracism. Soc Cogn Affect Neurosci.

[25] Sebastian CL (2011). Developmental influences on the neural bases of responses to social rejection: implications of social neuroscience for education. Neuroimage.

[26] Oaten M, Williams KD, Jones A, Zadro L (2008). The effects of ostracism on self-regulation in the socially anxious. J Soc Clin Psychol.

[27] Zadro L, Williams KD, Richardson R (2004). How low can you go?Ostracism by a computer is sufficient to lower self-reported levels of belonging, control, self-esteem, and meaningful existence. J Exp Soc Psychol.

[28] Gilboa-Schechtman E, Galili L, Sahar Y, Amir O (2014). Being “in” or “out” of the game: subjective and acoustic reactions to exclusion and popularity in social anxiety. Front Hum Neurosci.

[29] Freitas-Ferrari MC (2010). Neuroimaging in social anxiety disorder: a systematic review of the literature. Prog Neuropsychopharmacol Biol Psychiatry.

[30] Nishiyama Y (2015). fMRI Study of social anxiety during social ostracism with and without emotional support. PLoS ONE..

[31] Wakefield JC, Horwitz AV, Schmitz MF (2005). Are we overpathologizing the socially anxiou?Social phobia from a harmful dysfunction perspective. Can J Psychiatry.

[32] Brühl AB, Delsignore A, Komossa K, Weidt S (2014). Neuroimaging in social anxiety disorder: A meta-analytic review resulting in a new neurofunctional model. Neurosci Biobehav Rev.

[33] Maurage P (2012). Disrupted regulation of social exclusion in alcohol-dependence: An fMRI study. Neuropsychopharmacology.

[34] Liebowitz MR (1987). Social phobia. Mod Probl Pharmacopsychiatry.

[35] Beck, A. T., Steer, R. A. & Brown, G. K. *Beck Depression Inventory manual —second edition**.* (Psychological Corporation, 1996).

[36] Spielberger, C. D., Gorsuch, R. L., Lushene, R., Vagg P. R. & Jacobs G. *Manual for the state-trait anxiety inventory* (Consulting Psychologists Press, 1983).

[37] Poldrack RA (2007). Region of interest analysis for fMRI. Soc Cogn Affect Neurosci.

[38] Clark, D. M. & Wells, A. A cognitive model of social phobia In *Social phobia: Diagnosis, assessment, and treatment* (ed. Heimberg, R., Liebowitz, M., Hope, D. A. & Schneier, F. R.) 69–93 (Guilford Press, 1995).

[39] Servaas MN, Riese H, Ormel J, Aleman A (2014). The neural correlates of worry in association with individual differences in neuroticism. Hum Brain Mapp.

[40] Kühn S, Vanderhasselt M-A, De Raedt R, Gallinat J (2013). The neural basis of unwanted thoughts during resting state. Soc Cogn Affect Neurosci.

[41] Wittgenstein, L. *Philosophical Investigations* (Oxford: Blackwell, 1953).

[42] Davidson, D. Thought and talk in *Mind and Language*. (ed. Guttenplan, S.) (Oxford University Press, 1975).

[43] Shergill SS (2002). Modulation of activity in temporal cortex during generation of inner speech. Hum Brain Mapp.

[44] Morin A, Michaud J (2007). Self-awareness and the left inferior frontal gyrus: inner speech use during self-related processing. Brain Res Bull.

[45] Price M, Anderson PL (2011). The impact of cognitive behavioral therapy on post event processing among those with social anxiety disorder. Behav Res Ther.

[46] Cassin S (2011). & Rector, N. Mindfulness and the Attenuation of Post-Event Processing in Social Phobia: An Experimental Investigation. Cogn Behav Ther.

[47] McLean CP, Asnaani A, Litz BT, Hofmann SG (2011). Gender differences in anxiety disorders: Prevalence, course of illness, comorbidity and burden of illness. J Psychiatr Res.

[48] Xu X (2012). Gender differences in social anxiety disorder: Results from the national epidemiologic sample on alcohol and relations conditions. J Anxiety Disord.

[49] Zadro L, Williams KD, Richardson R (2004). How low can you go? Ostracism by a computer is sufficient to lower self-reported levels of belonging, control, self-esteem, and meaningful existence. J Exp Soc Psychol.

[50] Woo C-W, Krishnan A, Wager TD (2014). Cluster-extent based thresholding in fMRI analyses: Pitfalls and recommendations. Neuroimage.

[51] Friston KJ (1994). Assessing the significance of focal activations using their spatial extent. Hum Brain Mapp.

[52] Nichols TE (2012). Multiple testing corrections, nonparametric methods, and random field theory. Neuroimage.

[53] Brühl AB (2011). Neural correlates of altered general emotion processing in social anxiety disorder. Brain Res..

[54] Renier L (2013). Right occipital cortex activation correlates with superior odor processing performance in the early blind. PLoS ONE..

[55] Sheehan DV (1998). The Mini-International Neuropsychiatric Interview (M.I.N.I.): The development and validation of a structured diagnosis psychiatric interview for DSM-IV and ICD-10. J Clin Psychiatry..

[56] Bouvard, M. & Cottraux, J. Protocoles et échelles d’évaluation en psychiatrie et psychologie — 5^th^ edition (Masson, 2010).

[57] Heeren A (2012). The self-report version of the Liebowitz Social Anxiety Scale: Psychometric properties of the French version. Can J Behav Sci.

[58] Bruchon-Schweitzer, M. & Paulhan, I. *Adaptation francophone de l’inventaire d’anxiété Trait-Etat (Forme Y) de Spielberger* (Editions du Centre Psychologie Appliquée, 1993).

[59] Carp J (2012). The secret lives of experiments: methods reporting in the fMRI literature. Neuroimage.

[60] Hayasaka S, Nichols TE (2003). Validating cluster size inference: random field and permutation methods. Neuroimage.

[61] Friston, K.J. Experimental design and statistical issues In *Brain Mapping: The Disorders* (eds. Mazziotta, J.C., Toga, A.W., Frackowiak, R.S.J.). 33–59. (Academic Press, 2000).

[62] Heller R, Stanley D, Yekutieli D, Rubin N, Benjamini Y (2006). Cluster-based analysis of fMRI data. Neuroimage.

